# Predicting outcomes following endovascular aortoiliac revascularization using machine learning

**DOI:** 10.1038/s41746-025-01865-y

**Published:** 2025-07-24

**Authors:** Ben Li, Badr Aljabri, Derek Beaton, Leen Al-Omran, Mohamad A. Hussain, Douglas S. Lee, Duminda N. Wijeysundera, Ori D. Rotstein, Charles de Mestral, Muhammad Mamdani, Mohammed Al-Omran

**Affiliations:** 1 Department of Surgery, University of Toronto, Toronto, ON Canada; 2 Division of Vascular Surgery, St. Michael’s Hospital, Unity Health Toronto, University of Toronto, Toronto, ON Canada; 3 Institute of Medical Science, University of Toronto, Toronto, ON Canada; 4 Temerty Centre for Artificial Intelligence Research and Education in Medicine (T-CAIREM), University of Toronto, Toronto, ON Canada; 5 Department of Surgery, King Saud University, Riyadh, Saudi Arabia; 6 Data Science & Advanced Analytics, Unity Health Toronto, University of Toronto, Toronto, ON Canada; 7 College of Medicine, Alfaisal University, Riyadh, Saudi Arabia; 8 Division of Vascular and Endovascular Surgery and the Center for Surgery and Public Health, Brigham and Women’s Hospital, Harvard Medical School, Boston, MA USA; 9 Division of Cardiology, Peter Munk Cardiac Centre, University Health Network, Toronto, ON Canada; 10 Institute of Health Policy, Management and Evaluation, University of Toronto, Toronto, ON Canada; 11 ICES, University of Toronto, Toronto, ON Canada; 12 Department of Anesthesia, St. Michael’s Hospital, Unity Health Toronto, Toronto, ON Canada; 13 Li Ka Shing Knowledge Institute, St. Michael’s Hospital, Unity Health Toronto, Toronto, ON Canada; 14 Division of General Surgery, St. Michael’s Hospital, Unity Health Toronto, Toronto, ON Canada; 15 Leslie Dan Faculty of Pharmacy, University of Toronto, Toronto, ON Canada; 16 Department of Surgery, King Faisal Specialist Hospital and Research Center, Riyadh, Saudi Arabia

**Keywords:** Peripheral vascular disease, Atherosclerosis, Prognosis, Outcomes research

## Abstract

Endovascular aortoiliac revascularization is a common treatment option for peripheral artery disease that carries non-negligible risks. Outcome prediction tools may support clinical decision-making but remain limited. We developed machine learning algorithms that predict 30-day post-procedural outcomes. The National Surgical Quality Improvement Program targeted vascular database was used to identify patients who underwent endovascular aortoiliac revascularization between 2011–2021. Input features included 37 pre-operative demographic/clinical variables. The primary outcome was 30-day post-procedural major adverse limb event (MALE) or death. Data were split into training (70%) and test (30%) sets. Using 10-fold cross-validation, 6 machine learning models were trained using pre-operative features. Overall, 6601 patients were included, and 30-day MALE/death occurred in 470 (7.1%) individuals. The best-performing model was XGBoost, achieving an AUROC (95% CI) of 0.94 (0.93–0.95). In comparison, logistic regression had an AUROC (95% CI) of 0.74 (0.73–0.76). The XGBoost model accurately predicted 30-day post-procedural outcomes, performing better than logistic regression.

## Introduction

Peripheral artery disease (PAD) impacts more than 200 million people globally, leading to reduced quality of life, mounting health care costs, amputation, and death^1–4^. A distinct subset of PAD is aortoiliac occlusive disease (AIOD), which involves atherosclerosis of the infrarenal aorta and iliac arteries, affecting approximately 14% of the population in general^5^. Historically, advanced AIOD was treated with open revascularization^6^. Over the last few decades, endovascular intervention has become an increasingly common less invasive alternative^7^. Nevertheless, endovascular revascularization of AIOD carries a significant risk of adverse events, with a systematic review of 19 studies demonstrating mortality and complication rates up to 6.7% and 45%, respectively^8^. Consequently, careful assessment of perioperative risk is recommended by the Global Vascular Guidelines when patients are considered for revascularization^9^.

There are currently no standardized tools to support prediction of complications following endovascular revascularization for AIOD. The Vascular Quality Initiative (VQI) Cardiac Risk Index (CRI) is limited to open revascularization^10^. Other tools such as the American College of Surgeons (ACS) National Surgical Quality Improvement Program (NSQIP) online surgical risk calculator^11^ rely on modeling techniques that require manual input of clinical variables, making them challenging to use in busy medical environments^12^. Therefore, there is an important need to develop better and more practical risk prediction tools for patients undergoing endovascular aortoiliac revascularization.

Machine learning (ML) is a rapidly evolving technology that enables computers to learn from large amounts of data and make accurate predictions^13^. Using advanced analytics, ML can learn non-linear, complex relationships between user inputs, such as patient characteristics, and outputs, such as clinical outcomes^13^. The advantage of newer ML techniques over traditional statistical methods is that they can better model non-linear relationships between covariates and outcomes^14^, which is common in health care data^15^. Previously, we built a ML algorithm that can accurately predict 30-day complications following open revascularization for AIOD, which achieved better performance compared to logistic regression^16^. To complement this model and provide further guidance for the management of patients with advanced AIOD, NSQIP data were used to train ML algorithms that can predict 30-day outcomes following endovascular revascularization for AIOD.

## Results

### Patients and events

**Fig. 1:**
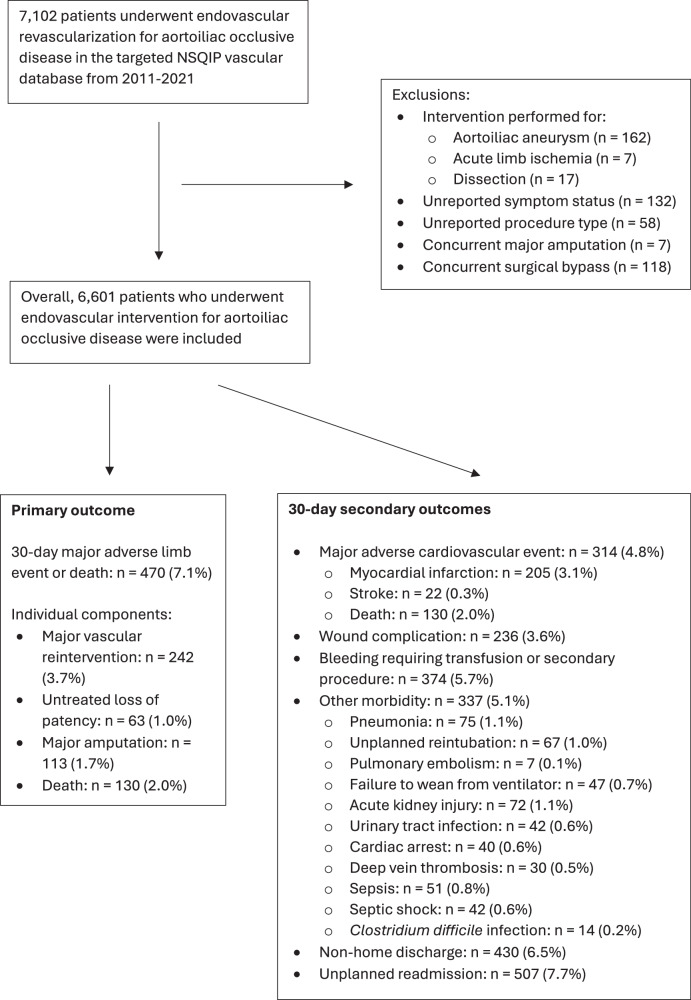
Flowchart for patient selection and outcomes. Patients undergoing open revascularization were not included in this study. NSQIP National Surgical Quality Improvement Program.

Overall, 7102 patients underwent endovascular revascularization for AIOD in the targeted NSQIP vascular database from 2011 to 2021. The following exclusions were made: intervention performed for aortoiliac aneurysm (*n* = 162), acute limb ischemia (*n* = 7), or dissection (*n* = 17), unreported symptom status (*n* = 132) or procedure type (*n* = 58), and concurrent major amputation (*n* = 7) or surgical bypass (*n* = 118). Overall, 6601 patients were included. The primary outcome of 30-day major adverse limb event (MALE) or death occurred in 470 (7.1%) individuals. The 30-day secondary endpoints occurred in this distribution: major vascular reintervention (*n* = 242 [3.7%]), untreated loss of patency (*n* = 63 [1.0%]), major amputation (*n* = 113 [1.7%]), death (*n* = 130 [2.0%]), major adverse cardiovascular event (MACE, *n* = 314 [4.8%]; composite of myocardial infarction (*n* = 205), stroke (*n* = 22), and death (*n* = 130)), wound complication (*n* = 236 [3.6%]), bleeding requiring transfusion or secondary procedure (*n* = 374 [5.7%]), other morbidity (*n* = 337 [5.1%]; composite of pneumonia (*n* = 75), unplanned reintubation (*n* = 67), pulmonary embolism (*n* = 7), failure to wean from ventilator (*n* = 47), acute kidney injury (*n* = 72), urinary tract infection (*n* = 42), cardiac arrest (*n* = 40), deep vein thrombosis (*n* = 30), sepsis (*n* = 51), septic shock (*n* = 42), *Clostridium difficile* infection (*n* = 14)), non-home discharge (*n* = 430 [6.5%]), and unplanned readmission (*n* = 507 [7.7%]). A flowchart summarizing patient selection and outcomes is reported in Fig. 1.

### Pre-operative characteristics

In comparison to patients who did not develop 30-day MALE or death, individuals with a primary outcome were older with a greater proportion being Black, residing in nursing homes or other facilities, or transferred from another hospital. A higher percentage of individuals with 30-day MALE or death had congestive heart failure (CHF), insulin-dependent diabetes, end stage renal disease (ESRD) requiring dialysis, and ≥1 high-risk physiologic factor, and were partially or totally dependent functionally. Despite having more cardiovascular comorbidities, a lower proportion of patients with an event received statins or antiplatelets. A greater proportion of patients with 30-day MALE or death had an ankle brachial index (ABI) ≤ 0.39 or no recorded ABI and pedal pulses that were non-palpable, as well as a prior surgical bypass involving the segment being treated. Individuals with a primary outcome were more likely to present with chronic limb threatening ischemia (CLTI), undergo more distal revascularization of the aortoiliac segment (i.e., external iliac rather than common iliac intervention), receive urgent or emergent intervention, and have an American Society of Anesthesiologists (ASA) classification ≥4. Patients who underwent intervention by vascular surgeons were less likely to develop a primary outcome compared to other specialists (Table 1).

**Table 1:** Pre-operative characteristics of patients undergoing endovascular aortoiliac revascularization with vs. without 30-day major adverse limb event or death

	Absence of 30-day MALE or death (*n* = 6131)	Presence of 30-day MALE or death (*n* = 470)	*P*
**Demographics**			
Age, years, mean (SD)	65.9 (10.7)	67.5 (11.1)	0.002
Female	2713 (44.3)	217 (46.2)	0.45
BMI, kg/m^2^, mean (SD)	27.8 (5.9)	26.7 (6.1)	< 0.001
Race			
White	4054 (66.1)	327 (69.6)	< 0.001
Black or African American	642 (10.5)	70 (14.9)	
Asian	34 (0.6)	4 (0.9)	
American Indian or Alaskan Native	20 (0.3)	0	
Native Hawaiian or Other Pacific Islander	2 (0.03)	2 (0.4)	
Other	2 (0.03)	0	
Unknown or not reported	1377 (22.5)	67 (14.3)	
Hispanic ethnicity	152 (2.5)	13 (2.8)	0.82
Origin			
Transferred from another hospital	322 (5.3)	84 (17.9)	< 0.001
Home	5736 (93.6)	365 (77.7)	
Nursing home	60 (1.0)	14 (3.0)	
Other facility	9 (0.1)	7 (1.5)	
Unknown	4 (0.07)	0	
Comorbidities			
Hypertension	4824 (78.7)	369 (78.5)	0.98
Diabetes			
Non-insulin dependent	1037 (16.9)	59 (12.6)	< 0.001
Insulin dependent	934 (15.2)	116 (24.7)	
Current smoker	3193 (52.1)	245 (52.1)	0.99
Congestive heart failure	194 (3.2)	38 (8.1)	< 0.001
Chronic obstructive pulmonary disease	955 (15.6)	89 (18.9)	0.06
Dialysis	179 (2.9)	41 (8.7)	< 0.001
Functional status			
Independent	5889 (96.1)	398 (84.7)	< 0.001
Partially dependent	215 (3.5)	65 (13.8)	
Totally dependent	12 (0.2)	5 (1.1)	
Unknown	15 (0.2)	2 (0.4)	
High-risk physiologic factor^a^	837 (13.7)	145 (30.9)	< 0.001
Medications			
Antiplatelet	4911 (80.1)	352 (74.9)	0.008
Statin	4423 (72.1)	306 (65.1)	0.001
Beta blocker	3117 (50.8)	268 (57.0)	0.01
Laboratory investigations			
Creatinine, umol/L, mean (SD)	102.0 (25.2)	127.0 (31.0)	< 0.001
BUN, mmol/L, mean (SD)	53.1 (29.0)	60.6 (37.9)	< 0.001
Sodium, mmol/L, mean (SD)	139.0 (3.1)	137.0 (4.0)	< 0.001
Hematocrit, L/L (%), mean (SD)	39.4 (5.6)	36.2 (6.6)	< 0.001
White blood cell count, cells/mm^3^, mean (SD)	8.4 (2.7)	10.0 (4.6)	< 0.001
Platelet count, 10^9^/L, mean (SD)	244.0 (79.3)	261.0 (102.0)	< 0.001
PTT, sec, mean (SD)	35.3 (11.2)	39.8 (16.4)	< 0.001
INR, mean (SD)	1.1 (0.2)	1.2 (0.4)	< 0.001
Albumin, g/L, mean (SD)	35.9 (4.0)	34.3 (5.6)	< 0.001
Anatomy/hemodynamics			
Limb hemodynamics			
ABI ≥ 1.3 and toe pressure < 30 mmHg	11 (0.2)	0	< 0.001
ABI ≥ 1.3 and toe pressure ≥ 30 mmHg	27 (0.4)	2 (0.4)	
ABI ≥ 1.3 and no toe pressure recorded	52 (0.8)	5 (1.1)	
ABI 0.90 – 1.29	278 (4.5)	9 (1.9)	
ABI 0.40 – 0.89	2458 (40.1)	118 (25.1)	
ABI ≤ 0.39	731 (11.9)	93 (19.8)	
ABI not recorded and pedal pulse not palpable	875 (14.3)	143 (30.4)	
ABI not recorded and palpable pedal pulse	296 (4.8)	14 (3.0)	
Not documented	1403 (22.9)	86 (18.3)	
High-risk anatomic factor			
Prior endovascular intervention involving currently treated segment	1198 (19.5)	88 (18.7)	0.003
Prior bypass involving currently treated segment	655 (10.7)	74 (15.7)	
None/not documented	4278 (69.8)	308 (65.5)	
Concurrent procedures			
Minor amputation	17 (0.3)	3 (0.6)	0.35
Infrainguinal endovascular revascularization	675 (11.0)	57 (12.1)	0.50
Other pre-procedural characteristics			
Symptom status			
Chronic limb threatening ischemia: tissue loss	918 (15.0)	176 (37.4)	< 0.001
Chronic limb threatening ischemia: rest pain	1247 (20.3)	155 (33.0)	
Claudication	3643 (59.4)	117 (24.9)	
Asymptomatic	323 (5.3)	22 (4.7)	
Primary procedure			
Aortic angioplasty/stent	182 (3.0)	16 (3.4)	< 0.001
Bilateral common iliac angioplasty/stent	1554 (25.3)	82 (17.4)	
Common iliac angioplasty/stent	1987 (32.4)	150 (31.9)	
External iliac angioplasty/stent	1417 (23.1)	138 (29.4)	
Internal iliac angioplasty/stent	72 (1.2)	1 (0.2)	
Common and external iliac angioplasty/stent	891 (14.5)	81 (17.2)	
Common and internal iliac angioplasty/stent	28 (0.5)	2 (0.4)	
Urgency			
Elective	5024 (81.9)	216 (46.0)	< 0.001
Urgent	859 (14.0)	171 (36.4)	
Emergent	247 (4.0)	83 (17.7)	
ASA class			
1	13 (0.2)	1 (0.2)	< 0.001
2	608 (9.9)	29 (6.2)	
3	3633 (59.3)	226 (48.1)	
4	1310 (21.4)	159 (33.8)	
5	4 (0.07)	7 (1.5)	
Not reported	563 (9.2)	48 (10.2)	
Specialty			
Vascular surgery	5684 (92.7)	431 (91.7)	0.001
Interventional radiology	253 (4.1)	33 (7.0)	
Other	194 (3.2)	6 (1.3)	

Values are reported as number (%) unless indicated otherwise. Missing data was < 5% for all variables of interest.

*ABI* ankle brachial index, *ASA* American Society of Anesthesiologists, *BMI* body mass index, *BUN* blood urea nitrogen, *INR* international normalized ratio, *MALE* major adverse limb event, *PTT* partial thromboplastin time, *SD* standard deviation.

^a^ ≥ 1 of the following: (1) end stage renal disease, (2) age over 80, (3) congestive heart failure class III/IV (New York Heart Association), (4) ejection fraction less than 30%, (5) unstable angina < 30 days before intervention, or (6) myocardial infarction < 30 days before intervention.

### Model performance

Six different ML models were trained and subsequently assessed on testing data for predicting 30-day MALE or death after endovascular revascularization for AIOD. Extreme Gradient Boosting (XGBoost) achieved the top performance with an area under the receiver operating characteristic curve [AUROC] (95% CI) of 0.94 (0.93–0.95) in comparison to random forest [0.92 (0.91–0.93)], radial basis function support vector machine [0.90 (0.89–0.91)], Naïve Bayes [0.85 (0.84–0.87)], multilayer perceptron artificial neural network [0.76 (0.74–0.77)], and logistic regression [0.74 (0.73–0.76)]. XGBoost attained the following secondary performance metrics: accuracy 0.86 (95% CI 0.85–0.88), specificity 0.87, sensitivity 0.86, negative predictive value 0.86, and positive predictive value 0.87 (Table 2).

**Table 2:** Model performance on testing data for prediction of 30-day major adverse limb event or death following endovascular aortoiliac revascularization using pre-operative features

	AUROC (95% CI)	Accuracy (95% CI)	Sensitivity	Specificity	PPV	NPV
XGBoost	0.94 (0.93–0.95)	0.86 (0.85–0.88)	0.86	0.87	0.87	0.86
Random forest	0.92 (0.91–0.93)	0.83 (0.82–0.85)	0.87	0.80	0.78	0.89
RBF SVM	0.90 (0.89–0.91)	0.82 (0.81–0.83)	0.81	0.83	0.84	0.80
Naïve Bayes	0.85 (0.84–0.87)	0.85 (0.84–0.87)	0.85	0.85	0.86	0.85
MLP ANN	0.76 (0.74–0.77)	0.70 (0.69–0.72)	0.70	0.71	0.73	0.67
Logistic regression	0.74 (0.73–0.76)	0.67 (0.65–0.69)	0.63	0.76	0.85	0.49

*AUROC* area under the receiver operating characteristic curve, *CI* confidence interval, *MLP ANN* multilayer perceptron artificial neural network, *NPV* negative predictive value, *PPV* positive predictive value, *RBF SVM* radial basis function support vector machine, *XGBoost* Extreme Gradient Boosting.

XGBoost obtained the following AUROC’s (95% CI) for predicting 30-day secondary endpoints: major vascular reintervention [0.86 (0.85–0.87)], untreated loss of patency [0.95 (0.94–0.96)], major amputation [0.97 (0.86–0.98)], death [0.97 (0.96–0.98)], MACE [0.90 (0.89–0.91)], bleeding requiring transfusion or secondary procedure [0.95 (0.94–0.96)], wound complication [0.90 (0.89–0.91)], other morbidity [0.88 (0.87–0.89)], non-home discharge [0.97 (0.96–0.98)], and unplanned readmission [0.88 (0.87–0.89)] (Table 3). Logistic regression had worse performance compared to XGBoost across all primary and secondary outcomes, with AUROC’s ranging from 0.70–0.78.

**Table 3:** XGBoost performance on testing data for prediction of 30-day primary and secondary outcomes following endovascular aortoiliac revascularization using pre-operative features

	AUROC (95% CI)	Accuracy (95% CI)	Sensitivity	Specificity	PPV	NPV
MALE or death	0.94 (0.93–0.95)	0.86 (0.85–0.88)	0.86	0.87	0.87	0.86
Major vascular reintervention	0.86 (0.85–0.87)	0.78 (0.77–0.80)	0.77	0.80	0.82	0.74
Untreated loss of patency	0.95 (0.94–0.96)	0.88 (0.87–0.89)	0.87	0.90	0.90	0.86
Major amputation	0.97 (0.86–0.98)	0.93 (0.92–0.94)	0.94	0.91	0.91	0.94
Death	0.97 (0.96–0.98)	0.92 (0.91–0.93)	0.92	0.91	0.91	0.92
MACE	0.90 (0.89–0.91)	0.82 (0.91–0.84)	0.82	0.83	0.83	0.81
Bleeding requiring transfusion or secondary procedure	0.95 (0.94–0.96)	0.89 (0.88–0.90)	0.89	0.89	0.89	0.88
Wound complication	0.90 (0.89–0.91)	0.82 (0.81–0.84)	0.81	0.84	0.85	0.80
Other morbidity	0.88 (0.87–0.89)	0.80 (0.79–0.82)	0.80	0.81	0.82	0.79
Non-home discharge	0.97 (0.96–0.98)	0.91 (0.90–0.92)	0.90	0.91	0.92	0.90
Unplanned readmission	0.88 (0.87–0.89)	0.80 (0.78–0.81)	0.79	0.80	0.81	0.78

*AUROC* area under the receiver operating characteristic curve, *CI* confidence interval, *MACE* major adverse cardiovascular event; composite of myocardial infarction, stroke, or death, *MALE* major adverse limb event; composite of untreated loss of patency, major vascular reintervention, or major amputation, *NPV* negative predictive value, *PPV* positive predictive value, *XGBoost* Extreme Gradient Boosting.

**Fig. 2:**
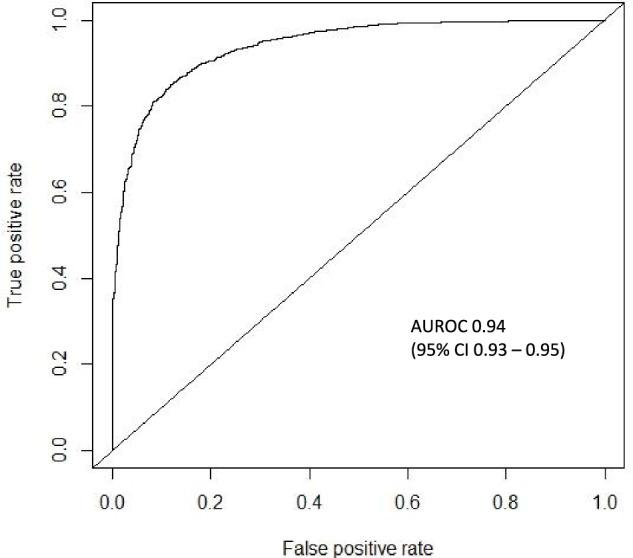
Receiver operating characteristic curve for predicting 30-day major adverse limb event or death following endovascular aortoiliac revascularization using Extreme Gradient Boosting (XGBoost) model. AUROC area under the receiver operating characteristic curve, CI confidence interval.

**Fig. 3:**
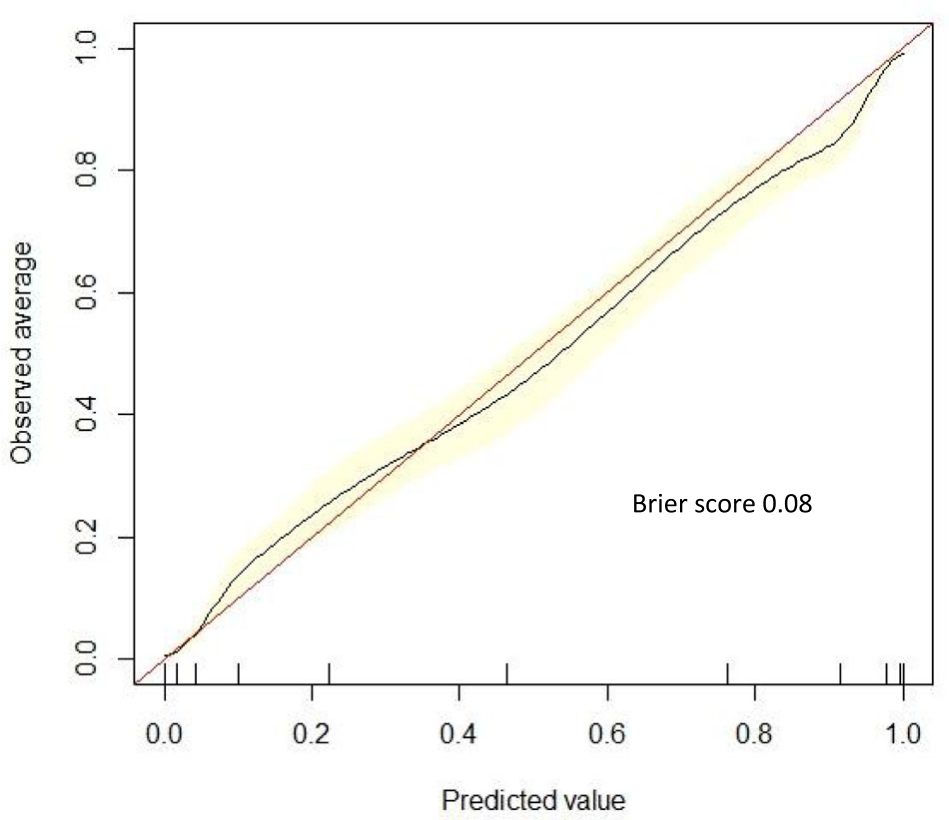
Calibration plot with Brier score. Extreme Gradient Boosting (XGBoost) model calibration for predicting 30-day major adverse limb event or death following endovascular aortoiliac revascularization.

**Fig. 4:**
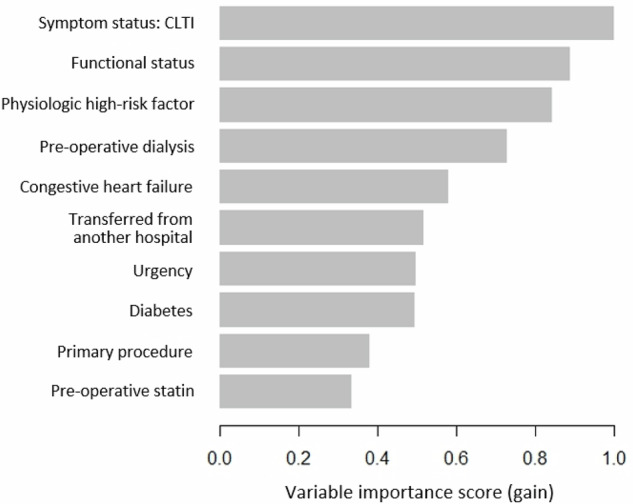
Variable importance scores (gain) for the top 10 predictors of 30-day major adverse limb event or death following endovascular aortoiliac revascularization in the Extreme Gradient Boosting (XGBoost) model. CLTI chronic limb threatening ischemia.

Figure 2 illustrates the ROC curve for XGBoost in predicting 30-day MALE or death. The model was well-calibrated, achieving a Brier score of 0.08, which demonstrates excellent agreement between predicted/observed event probabilities (Fig. 3). For XGBoost, the most important predictors of 30-day MALE or death were: (1) CLTI, (2) functional status, (3) ≥ 1 high-risk physiologic factor, (4) pre-operative dialysis, (5) CHF, (6) transferred from another hospital, (7) urgency, (8) diabetes, (9) primary procedure (more distal revascularization of the aortoiliac segment), and (10) absence of pre-operative statin (Fig. 4). Subgroup analysis of feature importance based on symptom status demonstrated that eight of the ten most important features were identical for CLTI patients and individuals who were asymptomatic or claudicants, and the two most important features were functional status and ≥1 high-risk physiologic factor for both subgroups (Supplementary Fig. 1).

### Subgroup analysis

Model performance was excellent across subgroups based on sex, age, race, ethnicity, procedure type, symptom status, urgency, and concurrent infrainguinal endovascular revascularization with a range of AUROC values from 0.92 to 0.95 and no significant differences were found between majority/minority subgroups (Supplementary Figs. 2–9).

## Discussion

Using targeted NSQIP vascular data from 2011 to 2021, which comprised 6601 patients who received endovascular revascularization for AIOD, we built ML algorithms that pre-operatively predict 30-day MALE or death with excellent performance (AUROC 0.94). Our models also predicted 30-day untreated loss of patency, major vascular reintervention, major amputation, death, MACE, wound complication, bleeding, other morbidity, non-home discharge, and unplanned readmission with AUROC’s ranging from 0.86 to 0.97. There were three other notable findings. First, individuals who suffer adverse events following endovascular aortoiliac revascularization are a high-risk cohort with predictive factors pre-operatively. They have more comorbidities and poorer functional status, with a greater proportion having high-risk anatomic and physiologic features. Furthermore, a greater proportion of patients with adverse events had CLTI, underwent more distal revascularization, and required urgent/emergent intervention. Despite these differences, they were less likely to receive optimal medical therapy including antiplatelets and statins. This represents an important opportunity to improve medical management of patients with PAD. Second, we evaluated six different ML models and XGBoost achieved the best predictive performance. Our algorithm was well-calibrated and remained robust across demographic/clinical subgroups. Third, we reported the most important predictors of 30-day MALE/death in our algorithms. These variables can help clinicians understand patient characteristics that lead to specific risk predictions, thereby supporting patient/procedure selection and pre-operative optimization.

Risk prediction tools for patients undergoing endovascular aortoiliac revascularization are limited. Bertges et al.^10^ used multivariable logistic regression to develop the VQI CRI for predicting myocardial infarction after carotid endarterectomy, lower extremity bypass, and aortic aneurysm repair, achieving an overall AUROC of 0.75^10^. Notably, the model did not include endovascular aortoiliac revascularization, and therefore, our work fills an important gap in this tool^10^. Applying ML to a contemporary NSQIP cohort, we achieved an AUROC of 0.94 for predicting MALE or death at 30 days following endovascular revascularization for AIOD. This demonstrates the benefit of leveraging advanced data analytics to build procedure-specific risk prediction models using contemporary datasets. Our model provides accurate risk predictions for a patient population that has often not been included in existing tools^10^ and complements our previously described ML algorithm for predicting outcomes at 30 days after open revascularization for AIOD^16^.

Bonde and colleagues (2021) built ML models on a sample of patients in the NSQIP database undergoing more than 2900 different procedures to predict post-operative complications, attaining AUROC’s between 0.85 and 0.88^17^. Since individuals with PAD are a high-risk population with many vascular comorbidities, the ability for general risk prediction tools to perform well on this cohort could be limited^18^. By building ML models tailored to individuals receiving endovascular revascularization for AIOD, we attained AUROC’s above 0.90. Our algorithms can also predict clinically relevant limb-related outcomes such as major vascular reintervention and major amputation, which are of importance to interventionalists and vascular surgeons. Consequently, developing ML algorithms specific to endovascular aortoiliac revascularization can increase performance and applicability.

Our findings can be interpreted in several ways. First, individuals who suffer from adverse events after endovascular revascularization for AIOD are a high-risk group with many cardiovascular risk factors^19^. Multiple societal guidelines recommend statins and antiplatelets for all PAD patients^9,20–22^, yet individuals who had complications in our study were less likely to be taking these medications. The BEST-CLI trial corroborates the fact that the rates of best medical therapy are suboptimal in PAD patients^23^. Consequently, there remain critical opportunities to improve outcomes for PAD patients by accurately determining their perioperative risk and optimizing them medically before intervention. In our ML algorithm, we found that clinical presentation with CLTI was the most important predictor of 30-day MALE or death, which corroborates existing literature given that these patients generally have more extensive lesions and severe ischemia, increasing the risk of limb loss and adverse events^24^. Poor pre-operative functional status was also a strong predictive feature for worse outcomes, as it correlates well with the overall health status of the patient and their ability to tolerate physiologically demanding procedures^25^. Other important predictors included high-risk physiological factors, pre-operative dialysis, pre-operative CHF, among others, highlighting that the overall clinical status of the patient and their pre-existing conditions are important predictors of their post-operative outcomes^26^. Second, the ML models achieved better performance than existing tools for various reasons. In comparison to logistic regression, newer ML technology can better learn non-linear and complex relationships between covariates and outcomes^27,28^. This is particularly important in health care data because many demographic/clinical factors can influence patient outcomes^29^. XGBoost was the top-performing algorithm, which has important advantages over other ML approaches including relatively fewer issues with overfitting in addition to quicker computing while retaining precision^30–32^. Additionally, XGBoost performs well on structured data, which could be the reason it outperformed more complicated algorithms such as neural networks^33^. These findings are corroborated by previous work demonstrating that ML algorithms can achieve better discrimination than conventional statistical models^34,35^. Additionally, we used methodology that strengthens model validity including calibration measurement, adherence to reporting standards (TRIPOD + AI statement), and collaboration between computer scientists, biostatisticians, and clinicians^34,35^. Third, the performance of our algorithms remained excellent across demographic/clinical subpopulations. This is notable given that algorithm bias against underrepresented populations remains an important concern within ML research^36^. These biases were likely avoided because ACS NSQIP is a multi-national database that captures diverse patient populations with rich sociodemographic information^37,38^. Fourth, a small proportion of patients in our cohort underwent revascularization for asymptomatic disease (~5%). The reasons for these interventions are unclear from our dataset but may be related to treatment of hemodynamically significant stenoses of previous revascularization procedures, patient preference, poor adherence to guideline-directed revascularization, or coding errors^39^.

Clinical decision-making can be supported by our ML algorithms in several ways. At the pre-operative stage, individuals predicted to be at elevated risk of adverse events could be assessed further in terms of both non-modifiable and modifiable factors^26^. Individuals with risks that are non-modifiable could be considered for alternative options including medical management alone, amputation, or close surveillance^40–42^. Those with modifiable risks could benefit from further evaluation and optimization including referrals to anesthesiologists, cardiologists, and/or internal medicine specialists when appropriate^43,44^. Conversely, low risk patients could be considered for open revascularization, which may be more durable^45^. Post-operatively, high risk patients could receive increased monitoring on the ward or intensive care unit^46^. Individuals at elevated risk for unplanned readmission or non-home discharge could receive increased support from allied health to ensure safe discharge planning^47^. These clinical decisions supported by the ML risk prediction tool may improve outcomes by mitigating complications related to endovascular revascularization for AIOD.

The ML algorithm programming code is openly available on GitHub. This tool may be utilized by clinicians caring for patients under consideration for endovascular revascularization for AIOD. The algorithm could be implemented at the >700 NSQIP-participating centers globally. There is also potential to implement the model at non-NSQIP sites because the input variables are commonly captured for the routine care of vascular patients^48^. Given the challenges of deploying prediction models into clinical practice, consideration of implementation science principles is critical^49^. Our ML models have the advantage of providing automated risk predictions, thereby improving practicality in busy clinical settings compared with traditional risk predictors that generally require manual input of variables^11^. Specifically, our algorithm can autonomously extract a patient’s NSQIP data to make risk predictions. The clinical applicability of our risk prediction tool stems from both its automated nature and highly accurate predictions that can guide vascular specialists in terms of patient/procedure selection, counseling, and peri-operative management to improve care for patients with AIOD being considered for endovascular revascularization. We advocate for dedicated health care data analytics teams at the institution level, as their benefits to patient care have been previously demonstrated and model implementation can be facilitated by these experts^50^.

Our study has various limitations. First, the algorithms were built using data from NSQIP. Additional studies are needed to evaluate whether generalization of predictive performance can be made to non-NSQIP centers. Second, our dataset captured 30-day endpoints. Assessment of ML algorithms on datasets with longer follow-up periods could support prediction of long-term risk. Third, several variables that may contribute to risk predictions were not captured in our dataset, including lesion characteristics and use of low-dose rivaroxaban, drug eluting technology, post-procedural antiplatelet therapy, intravascular lithotripsy, covered stents, and intravascular ultrasound. Future predictive models developed on datasets that record these variables could improve accuracy. Additionally, some data points were unknown, not reported, or not documented; further refinement of datasets with more complete clinical information may improve model performance. Fourth, the sample size was lower than expected over a 10-year period likely because ACS NSQIP is primarily a surgical database, and procedures performed by interventional radiologists or other non-surgical specialists may be under-captured. Additional investigation of endovascular aortoiliac revascularization performed by non-surgical specialists may increase the sample size for analysis.

Using the NSQIP targeted vascular database, we built ML algorithms that predict 30-day MALE/death and other clinically relevant outcomes following endovascular revascularization for AIOD with excellent performance using pre-operative data. Given that our ML algorithms performed better than existing tools and logistic regression, they have potential for important utility in the peri-operative management of patients being considered for endovascular aortoiliac revascularization to mitigate adverse outcomes. Prospective validation of our prediction models is warranted.

## Methods

### Design

This was an ML-based prognostic study and findings were reported based on the Transparent Reporting of a Multivariable Prediction Model for Individual Prognosis or Diagnosis + Artificial Intelligence (TRIPOD + AI) statement^51^. The methods were based on our previous work to demonstrate the robustness and reproducibility of our ML model development and evaluation process^16^.

### Dataset

The ACS NSQIP database contains data on patients from >700 hospitals in ~15 countries worldwide^52^. Trained clinical reviewers prospectively collect data from electronic health records and ACS regularly audits the data for accuracy^53^. Targeted vascular registries in NSQIP were established in 2011 and comprise additional variables and outcomes specific to vascular procedures^54^. Research ethics board review was not required as the data source was a deidentified registry.

### Cohort

All individuals in the NSQIP targeted vascular database who received endovascular revascularization for AIOD (angioplasty or stent of the aorta or iliac arteries) for PAD between 2011 and 2021 were included. Individuals who underwent the procedure for aortoiliac aneurysm, dissection, acute limb ischemia, malignancy, or trauma, patients with unreported symptom status or procedure type, and those who received concurrent major amputation or bypass were excluded.

### Features

The input features for the ML models included 37 pre-operative variables. To maximize model performance, all pre-operative NSQIP variables were used, as ML methods excel at handling many inputs. Feature selection was not performed as it worsened model performance, and given the goal of automated predictions, reducing the number of features would decrease predictive performance without a significant change to model run time. Demographic features included age, body mass index, sex, race, and ethnicity. Comorbidities consisted of diabetes, hypertension, smoking status, chronic obstructive pulmonary disease, CHF, ESRD on dialysis, functional status, and high-risk physiologic factor [defined as ≥1 of: (1) ESRD, (2) age above 80, (3) CHF class III or IV (New York Heart Association), (4) ejection fraction below 30%, (5) unstable angina <30 days before intervention, or (6) myocardial infarction <30 days before intervention]. Medications consisted of statins, antiplatelets, and beta blockers. Labs included serum creatinine, blood urea nitrogen, serum sodium, albumin, hematocrit, white blood cell count, platelet count, partial thromboplastin time, and international normalized ratio. Limb hemodynamics based on ABI and toe pressure, and high-risk anatomic factors (defined as a prior bypass or endovascular intervention involving the currently treated segment), were noted. Concurrent procedures documented were minor amputation (below the ankle) and endovascular infrainguinal revascularization. Other characteristics were symptom status [CLTI (defined as tissue loss or rest pain), claudication, or asymptomatic], procedure type, urgency of intervention (elective, urgent, or emergent), ASA class, and specialty of the primary operator. Urgent and emergent interventions, generally performed within 24–48 h and <24 h of admission, respectively, were mostly performed for patients with severe ischemia resulting in CLTI. Isolated internal iliac artery interventions were included and generally performed on patients with lifestyle-limiting buttock claudication, rest pain, and/or tissue loss. Supplementary Table 1 provides a complete list of input features and definitions.

### Outcomes

The primary endpoint was 30-day post-procedural MALE (defined as a composite of major vascular reintervention, untreated loss of patency, or major amputation) or death. The definition of major vascular reintervention was thrombectomy/thrombolysis involving the treated segment or a new or revision aortoiliac bypass or interposition graft. The definition of untreated loss of patency was an occlusion of the treated segment with no subsequent revascularization. The definition of major amputation was a transtibial or more proximal amputation of the ipsilateral leg. The definition of death was all-cause mortality. These outcomes and definitions align with the Society for Vascular Surgery reporting standards^55^.

The 30-day secondary outcomes consisted of individual components of MALE or death, MACE, bleeding requiring transfusion or secondary procedure, wound complication, other morbidity, non-home discharge, and unplanned readmission. MACE was a composite endpoint of myocardial infarction (ischemic changes on electrocardiogram, elevated troponin, or diagnosis by a physician or advanced provider), stroke (neurologic dysfunction exceeding 24 h in the setting of suspected stroke), or death. The definition of wound complication was a non-healing wound at the incision (if present), cellulitis, or dehiscence. Other morbidity was a composite endpoint of unplanned reintubation, failure to wean from the ventilator (cumulative time of ventilator-assisted respirations over 48 h), pneumonia, pulmonary embolism, acute kidney injury (rise in serum creatinine over 2 mg/dL from the pre-operative value or requirement of dialysis in a patient who was not on dialysis pre-operatively), cardiac arrest, urinary tract infection, *Clostridium difficile* infection, deep vein thrombosis requiring therapy, sepsis, or septic shock. The definition of non-home discharge was discharge to skilled care, rehabilitation, or other facility. These outcomes are defined by the ACS NSQIP data dictionary^56^.

### Model development

We trained 6 ML models to predict 30-day primary and secondary outcomes: Naïve Bayes classifier, XGBoost, radial basis function support vector machine, random forest, multilayer perceptron artificial neural network, and logistic regression. We selected these models because of their demonstrated efficacy in predicting postoperative outcomes^57–59^. We used logistic regression for baseline comparison because traditional risk predictors most commonly use this modeling technique^60^.

We randomly split our data into two subsets: 70% training and 30% testing^61^. Testing data was reserved for model evaluation and not used for model training. We performed 10-fold cross-validation and grid search on training data to identify hyperparameters that were optimized for each ML model, including logistic regression^62,63^. The multivariable logistic regression model was developed and optimized with the same rigor as the advanced ML models with 10-fold cross-validation and grid search^62,63^. Preliminary analysis showed that the primary endpoint was rare, affecting 470/6601 (7.1%) of patients. To improve class balance, Random Over-Sample Examples (ROSE) was applied to training data^64^. ROSE uses smoothed bootstrapping to draw new samples from the feature space neighborhood around the minority class and is a commonly used method to facilitate predictive modeling of rare events^64^. The models were then assessed on test set data and ranked based on the primary discriminatory metric of AUROC. Our best performing model was XGBoost, which had the following optimized hyperparameters for our dataset: number of rounds = 200, maximum tree depth = 3, learning rate = 0.3, gamma = 0, column sample by tree = 0.6, minimum child weight = 1, and subsample = 0.9. The process for selecting these hyperparameters is described in Supplementary Table 2. Once the top-performing ML algorithm for the primary endpoint was identified, the same model was further trained to predict secondary endpoints.

### Statistical analysis

Baseline features were reported as means and standard deviations or numbers and proportions. Differences between patients with vs. without 30-day MALE or death were evaluated with independent *t*-test (continuous variables) or chi-square test (categorical variables). All continuous data were normally distributed. To account for multiple comparisons, Bonferroni correction was used to set statistical significance. AUROC was the primary model evaluation metric as it considers both sensitivity and specificity^65^. Secondary model evaluation metrics were specificity, sensitivity, negative predictive value, positive predictive value, and accuracy. Model robustness was evaluated using a calibration curve and Brier score, which measures the agreement between predicted/observed event probabilities^66^. Feature importance was assessed using the variable importance score (gain), which measures the influence of each covariate in contributing to a prediction^67^. We reported feature importance for 3 groups: the overall cohort, asymptomatic/claudication patients, and individuals with CLTI. Model performance was assessed across subgroups based on sex, age, race, ethnicity, procedure type, symptom status, urgency, and concurrent infrainguinal endovascular revascularization.

Using a sample size calculator validated for prediction models, we determined that to achieve an AUROC over 0.8 with an outcome rate of approximately 7% and 37 input variables, 3815 patients with 268 events was the minimum cohort size required^68,69^. We included 6601 patients who had 470 primary events, which satisfied this condition. There was < 5% missing data for variables of interest; therefore, available case analysis was applied whereby only non-missing covariates for each patient were considered^70,71^. This is a valid method for analyzing datasets with minimal missing data^70,71^. Data were missing completely at random, and imputation was not performed to reflect modeling of real-world data, which generally has missing information that is not imputed^72^. All subjects included in the study (*n* = 6601) were used in all analyses. R version 4.3.0^73^ was used for all analyses with the following packages: caret^74^, xgboost^75^, ranger^76^, naivebayes^77^, e1071^78^, nnet^79^, and pROC^80^.

## Supplementary information

Supplementary Information

## Data Availability

The data used for this study comes from the American College of Surgeons National Surgical Quality Improvement Program (ACS NSQIP). Access to and use of the data requires approval through an application process available at https://www.facs.org/quality-programs/data-and-registries/acs-nsqip/participant-use-data-file/.

## Code availability

The complete code used for model development and evaluation in this project is openly available on GitHub: https://github.com/benli12345/AIE-ML-NSQIP.
