# Supplementary material for: Predicting outcomes following endovascular aortoiliac revascularization using machine learning
Source: NPJ Digit Med. 2025 Jul 24;8:475. doi: 10.1038/s41746-025-01865-y (PMC12289885; doi:10.1038/s41746-025-01865-y)
Supplement: Supplementary file 1 — Supplementary Information [file 41746_2025_1865_MOESM1_ESM.pdf]

Supplementary Table 1. Pre-operative input features for machine learning models

**Features (n = 37)**	**Definition based on ACS NSQIP manual**
**Logistics**	
Admission quarter	Quarter of hospital admission
Operation year	Year procedure was performed
**Demographics**	
Age	Age (years)
Body mass index	Weight in kg / height in m^2
Sex	Male or female
Race	As per medical record or self-assigned by the patient: 1) White, 2) Black or African American, 3) American Indian or Alaskan Native, 4) Native Hawaiian or Other Pacific Islander, 5) Asian, 6) Other, 7) Unknown/not reported
Ethnicity	Hispanic or non-Hispanic
Origin status	1) Transferred from another hospital for any reason, including transferring hospital not having vascular surgery/interventional capabilities, complex cases for tertiary care, or urgent repair, or from 2) home, 3) nursing home, 4) other facility, or 5) unknown
**Comorbidities**	
Hypertension	Diagnosis documented in medical record and patient requires antihypertensive medication within 30 days prior to surgery
Current smoker	Patient has smoked cigarettes at any point within the 12 months prior to surgery
Diabetes	Documented diagnosis; non-insulin dependent or insulin-dependent
Chronic obstructive pulmonary disease	Diagnosis documented in medical record and at least 1 of the following within 30 days prior to surgery: 1) functional disability from chronic obstructive pulmonary disease, 2) requires chronic bronchodilator therapy, 3) hospitalization at any time in the past for treatment of chronic obstructive pulmonary disease, or 4) a forced expiratory volume (FEV) 1 of < 75% on any prior pulmonary function test
Congestive heart failure	Diagnosis documented by physician or advanced provider within 30 days prior to surgery and documentation of at least 1 of the following within 30 days prior to surgery: 1) active signs or symptoms of congestive heart failure, 2) New York Heart Association Functional Classification II-IV, 3) daily prescription of disease-modifying drugs for heart failure, 4) left ventricular ejection fraction < 40% on the most recent measurement prior to surgery, 5) elevated levels of natriuretic peptides (BNP ≥100 pg/mL or NT-proBNP ≥900 pg/mL), 6) patients with one of the following devices: ventricular assist device, implantable cardioverter defibrillator, or cardiac resynchronization therapy device.

**Features (n = 37)**	**Definition based on ACS NSQIP manual**
Dialysis	Documented peritoneal dialysis, hemodialysis, hemofiltration, hemodiafiltration, or ultrafiltration within 2 weeks prior to surgery
Physiologic high-risk factor	At least 1 of the following: 1) end stage renal disease, 2) age > 80, 3) New York Heart Association congestive heart failure class III/IV, 4) left ventricular ejection fraction < 30%, 5) unstable angina within 30 days prior to surgery, or 6) myocardial infarction within 30 days prior to surgery
Functional status	1) Independent (does not require assistance for activities of daily living), 2) partially dependent (requires some assistance for activities of daily living), 3) totally dependent (requires total assistance for all activities of daily living, or 4) unknown
**Medications**	Patient was on medication when considered for intervention or when intervention was decided for the patient
Statin	Includes atorvastatin, simvastatin, fluvastatin, lovastatin, pitavastatin, rosuvastatin, and pravastatin
Antiplatelet	Includes aspirin, clopidogrel, eptifibatide, and aggrenox
Beta blocker	Includes acebutolol, atenolol, betaxolol, bisoprolol, carvedilol, esmolol, labetalol, metoprolol, nadolol, nebivolol, pindolol, propranolol, sotalol, andtimolol
**Pre-operative laboratory investigations**	Collected within 90 days prior to intervention
Serum creatinine	Reported in umol/L
Blood urea nitrogen	Reported in mmol/L
Serum sodium	Reported in mmol/L
Hematocrit	Reported in %
White blood cell count	Reported in cells/mm^3
Platelet count	Reported in 10^9/L
PTT	Reported in seconds
INR	Unitless ratio
Albumin	Reported in g/L
**Anatomy/hemodynamics**	
Limb hemodynamics	1) ABI ≥ 1.3; OR if arteries are described as "noncompressible" AND the toe pressure ≥ 30 mm Hg<br>2) ABI ≥ 1.3; OR if arteries are described as "noncompressible" AND toe pressure < 30 mm Hg<br>3) ABI ≥ 1.30; OR if arteries "noncompressible", no toe pressure taken<br>4) ABI 0.9- 1.29<br>5) ABI 0.4-0.89<br>6) ABI ≤ 0.39<br>7) ABI not performed AND ipsilateral pedal pulse is palpable<br>8) ABI not performed AND ipsilateral pedal pulse is non-palpable<br>9) None/Not documented

**Features (n = 37)**	**Definition based on ACS NSQIP manual**
Anatomic high-risk factor	1) Prior ipsilateral bypass involving the currently treated segment, 2) prior ipsilateral endovascular intervention involving the currently treated segment, or 3) none/not documented
**Concurrent procedures**	Procedure occurring in the same setting as the index surgery
Infrainguinal endovascular intervention	Endovascular intervention on arterial segment below the inguinal ligament
Minor amputation	Amputation below the level of the ankle
**Other pre-procedural characteristics**	
Primary procedure	1) Aortic angioplasty/stent<br>2) Bilateral common iliac angioplasty/stent<br>3) Common iliac angioplasty/stent<br>4) External iliac angioplasty/stent<br>5) Internal iliac angioplasty/stent<br>6) Common and external iliac angioplasty/stent<br>7) Common and internal iliac angioplasty/stent
Symptom status	1) Asymptomatic, 2) claudication, 3) chronic limb threatening ischemia: rest pain, or 4) chronic limb threatening ischemia: tissue loss
ASA class	1) normal healthy patient, 2) mild systemic disease, 3) severe systemic disease, 4) severe systemic disease that is a constant threat to life, 5) moribund patient who is not expected to survive without the operation, or 6) not reported
Urgency	Elective, urgent, or emergent as per documentation by the surgeon, anesthesiologist, nurse, or case schedule
Specialty	Specialty of the primary operator: 1) vascular surgeon, 2) interventional radiologist, or 3) other

Abbreviations: ABI (ankle brachial index), ASA (American Society of Anesthesiologists), BNP (brain natriuretic peptide), INR (international normalized ratio), PTT (partial thromboplastin time).

Supplementary Table 2. Hyperparameter selection for Extreme Gradient Boosting (XGBoost) model using grid search and cross validation

Hyperparameter	Values tested through grid search and cross validation*	Optimal value chosen to maximize AUROC
Learning rate	0.4, 0.3, 0.2, 0.1, 0.05, 0.01, 0.001	0.3
Maximum tree depth	2, 3, 4, 5, 6, 7, 8, 9	3
Number of rounds	50, 100, 150, 200, 250, 300, 350, 400, 450, 500	200
Column sample by tree	0.5, 0.6, 0.7, 0.8, 0.9, 1	0.6
Subsample	0.5, 0.6, 0.7, 0.8, 0.9, 1	0.9
Minimum child weight	1, 3, 5, 7, 10	1
Gamma	0, 0.1, 1, 1.5, 2	0

Abbreviation: AUROC (area under the receiver operating characteristic curve).

*Grid search and cross validation are exhaustive methods that iteratively train and evaluate models using every combination of specified hyperparameter values and selects the set of hyperparameter values that optimize model performance.

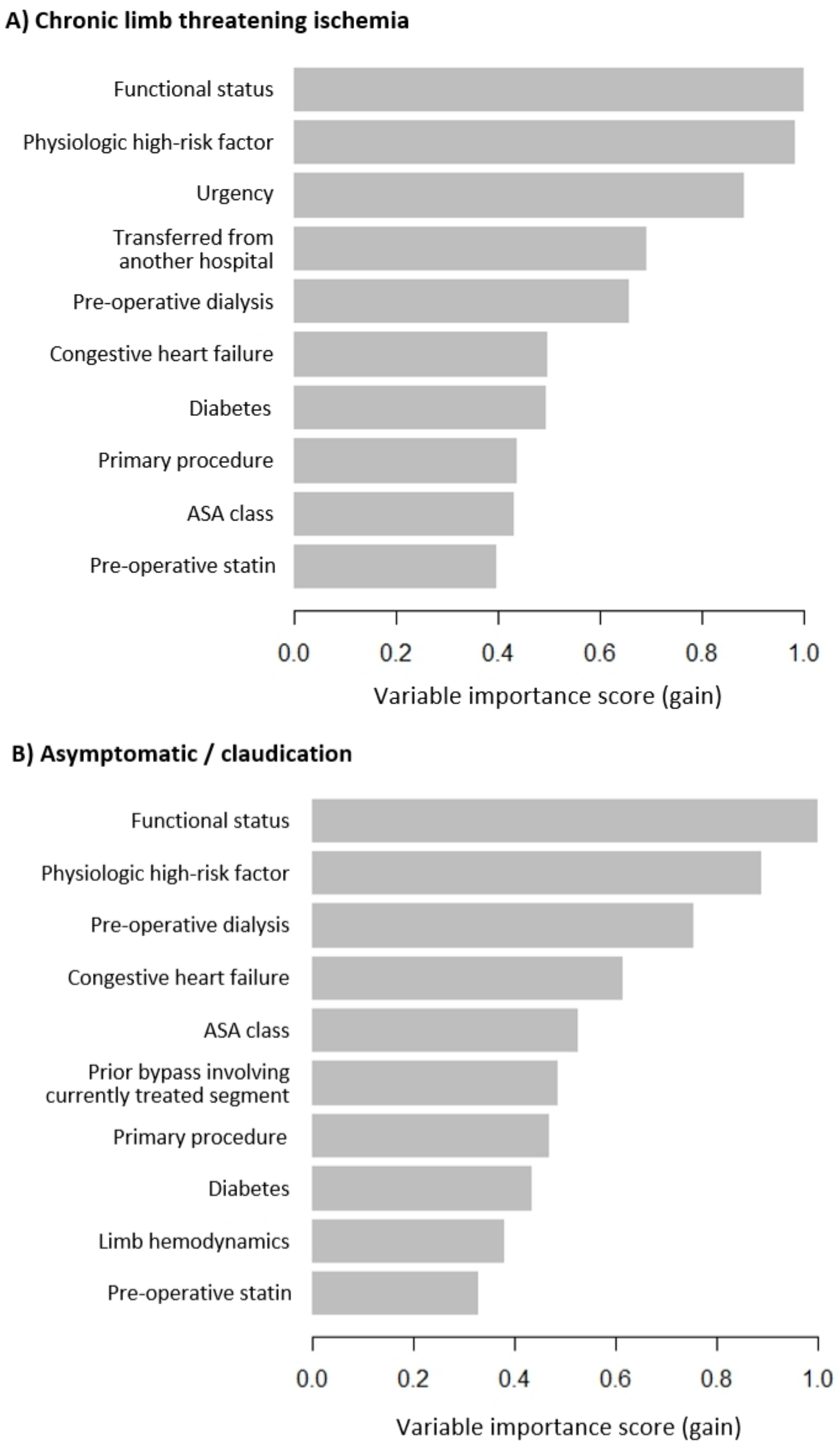

Supplementary Figure 1. Variable importance scores for the top 10 predictors of 30-day major adverse limb events or death after endovascular aortoiliac revascularization in the Extreme Gradient Boosting (XGBoost) model with subgroup analysis based on symptom status: A) chronic limb threatening ischemia and B) asymptomatic / claudication. Abbreviation: ASA (American Society of Anesthesiologists).

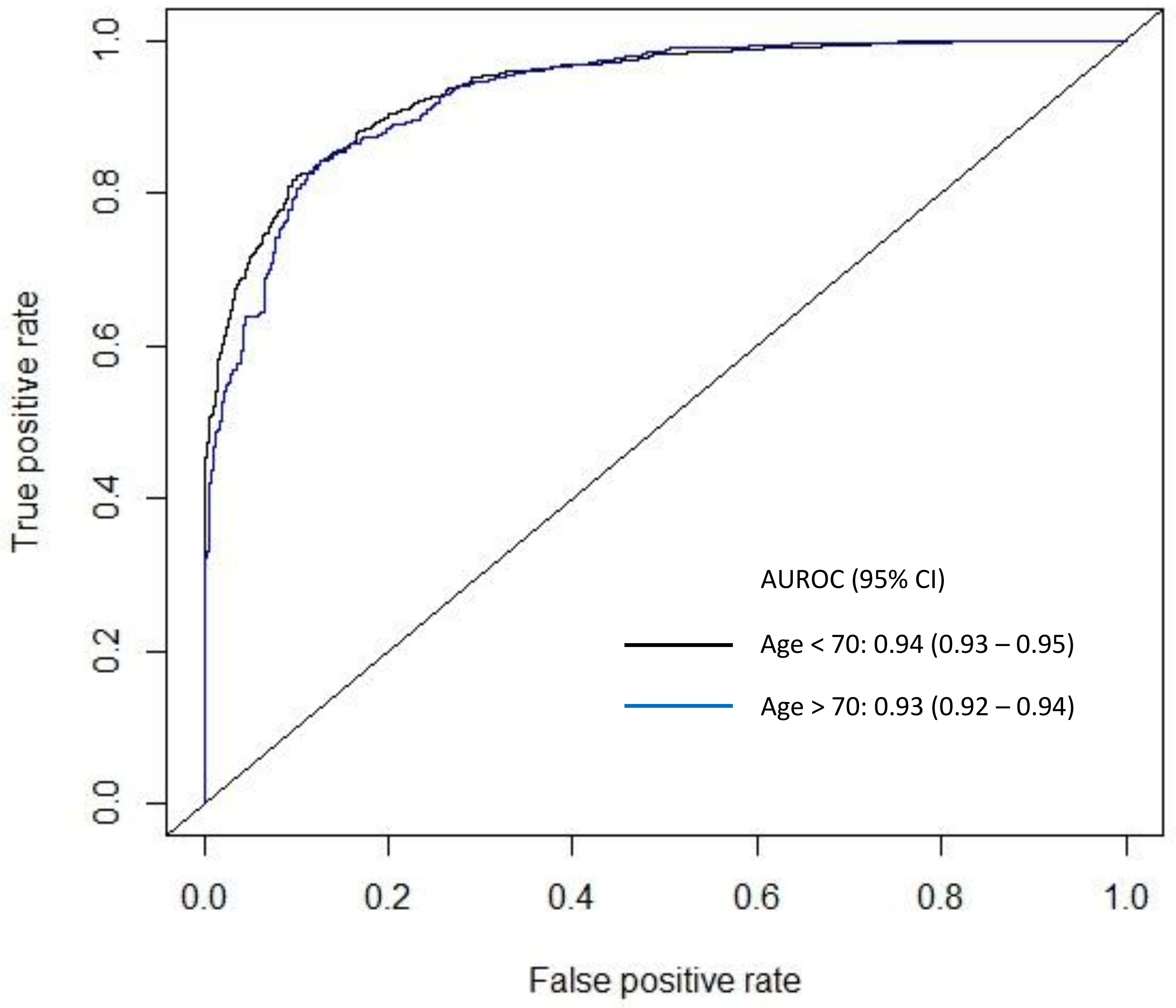

Supplementary Figure 2. Receiver operating characteristic curve for Extreme Gradient Boosting (XGBoost) model in predicting 30-day major adverse limb event or death following endovascular aortoiliac revascularization with subgroup analysis based on age. AUROC (area under the receiver operating characteristic curve), CI (confidence interval).

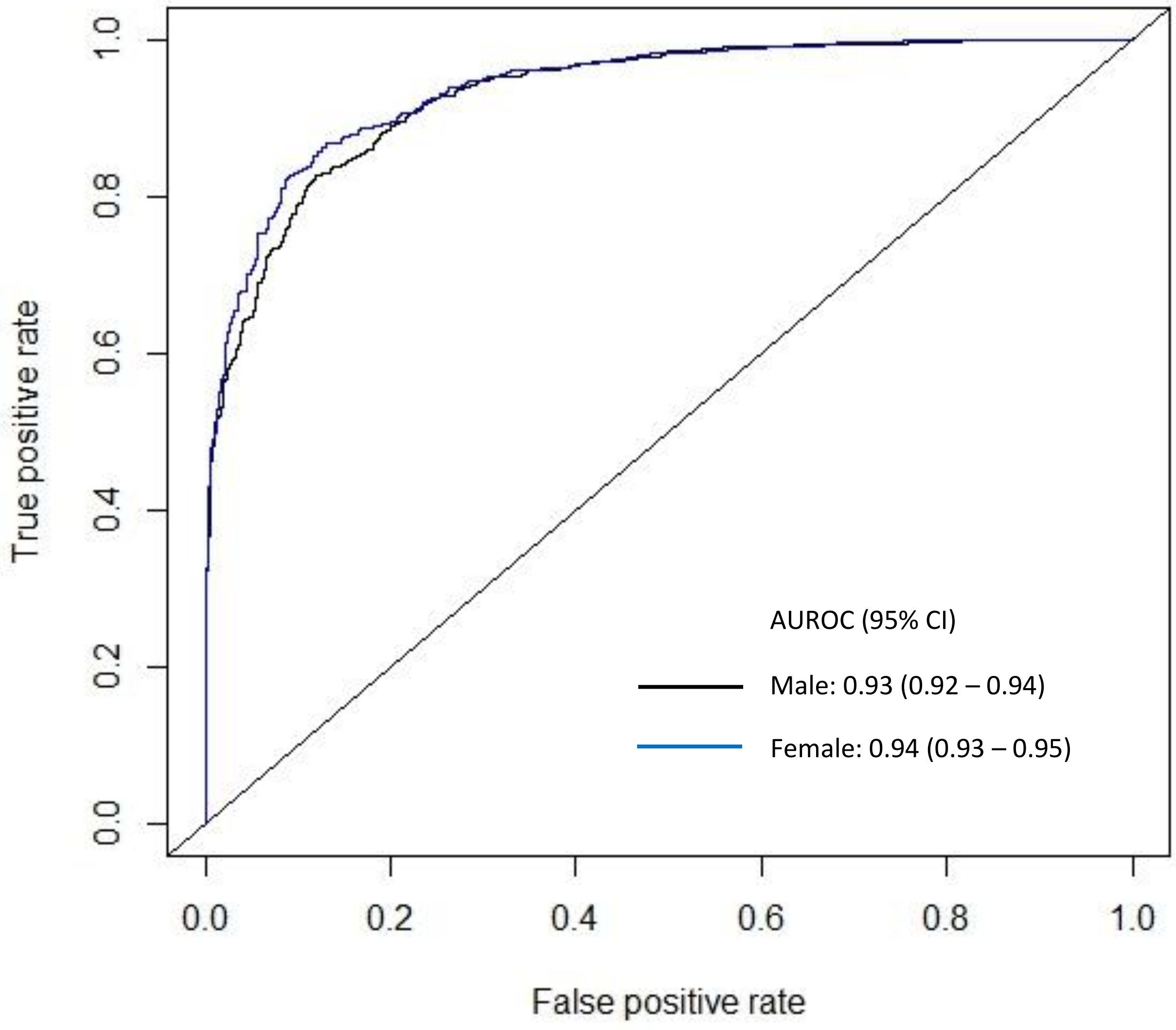

Supplementary Figure 3. Receiver operating characteristic curve for Extreme Gradient Boosting (XGBoost) model in predicting 30-day major adverse limb event or death following endovascular aortoiliac revascularization with subgroup analysis based on sex. AUROC (area under the receiver operating characteristic curve), CI (confidence interval).

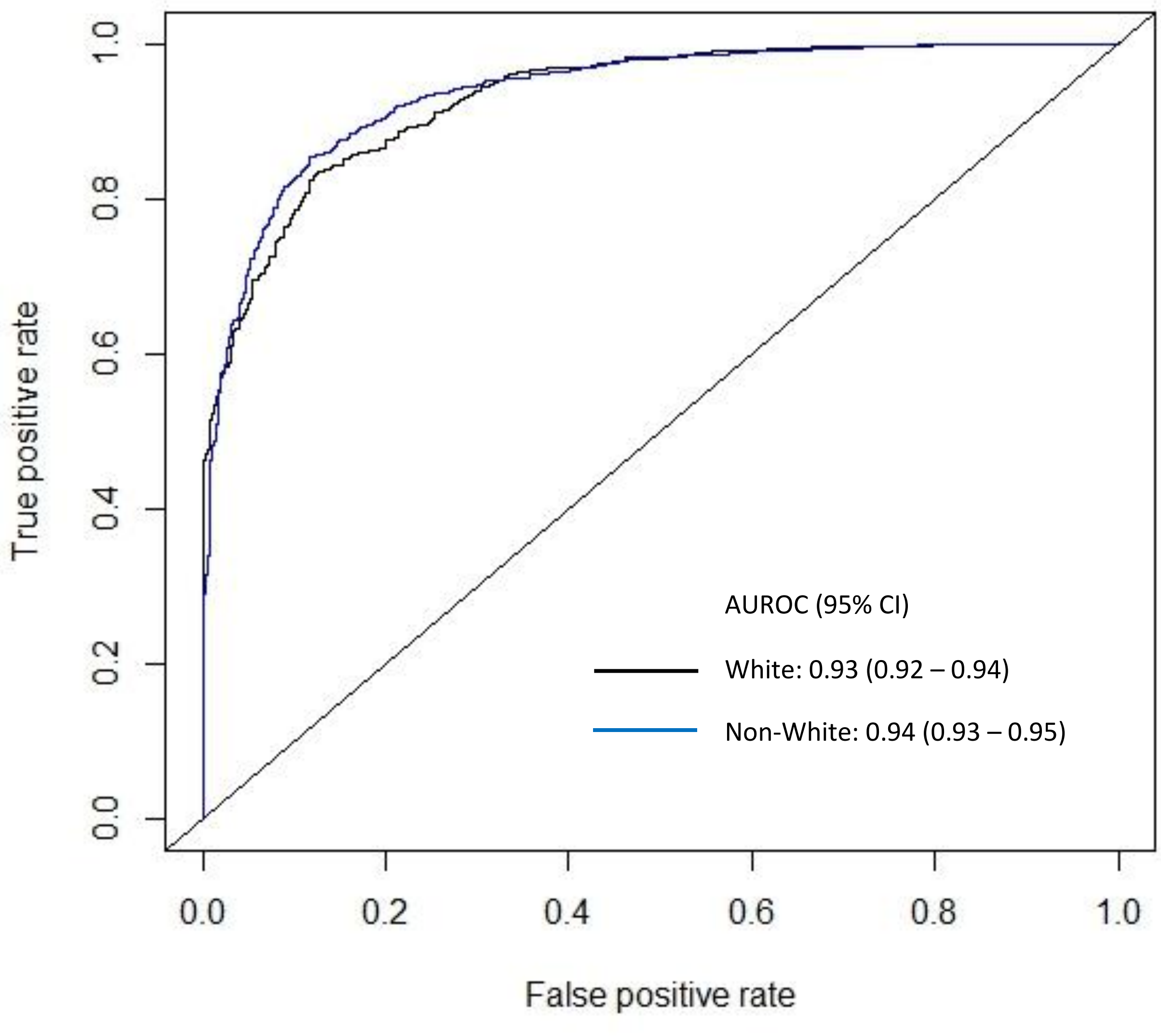

Supplementary Figure 4. Receiver operating characteristic curve for Extreme Gradient Boosting (XGBoost) model in predicting 30-day major adverse limb event or death following endovascular aortoiliac revascularization with subgroup analysis based on race. AUROC (area under the receiver operating characteristic curve), CI (confidence interval).

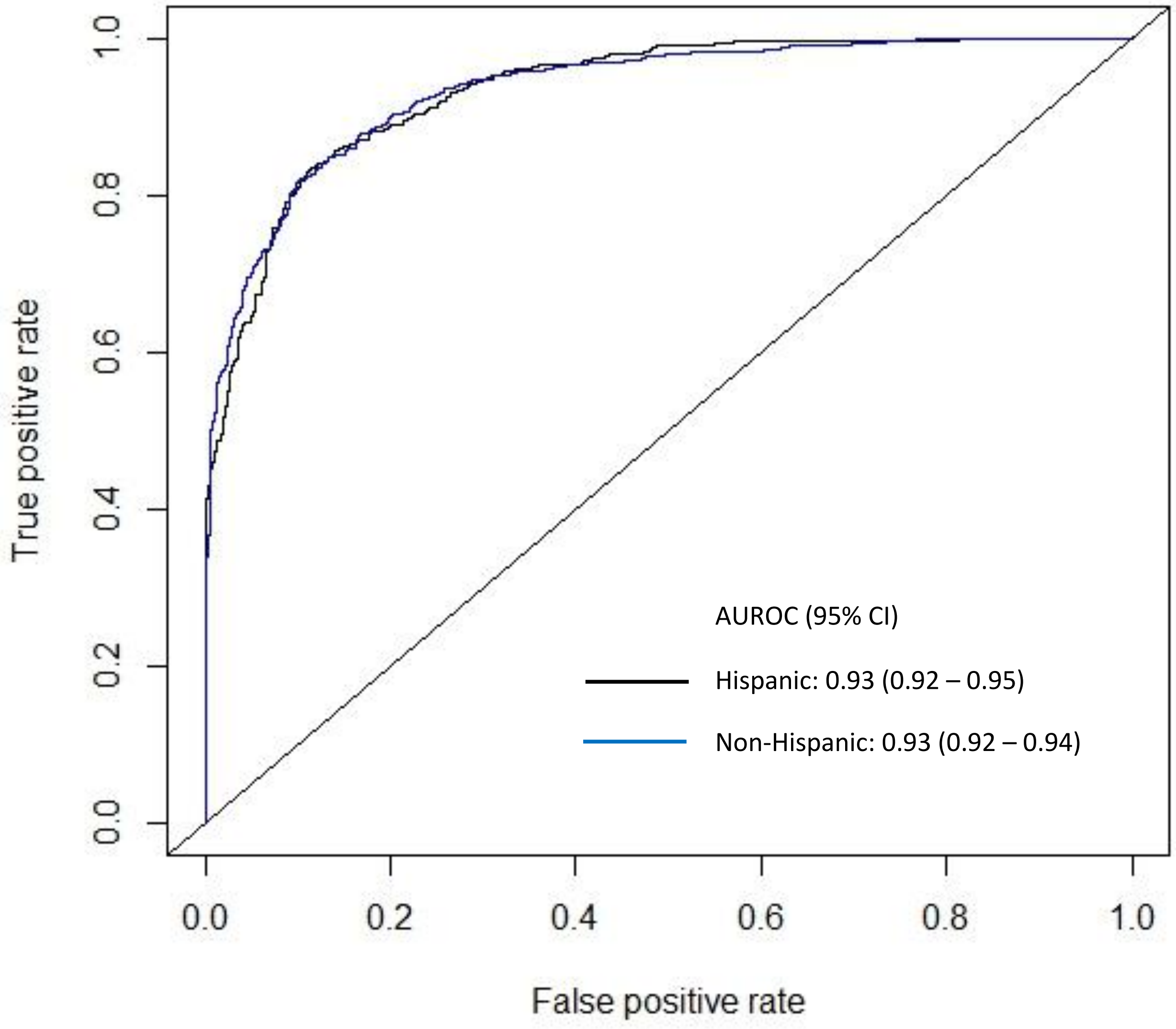

Supplementary Figure 5. Receiver operating characteristic curve for Extreme Gradient Boosting (XGBoost) model in predicting 30-day major adverse limb event or death following endovascular aortoiliac revascularization with subgroup analysis based on ethnicity. AUROC (area under the receiver operating characteristic curve), CI (confidence interval).

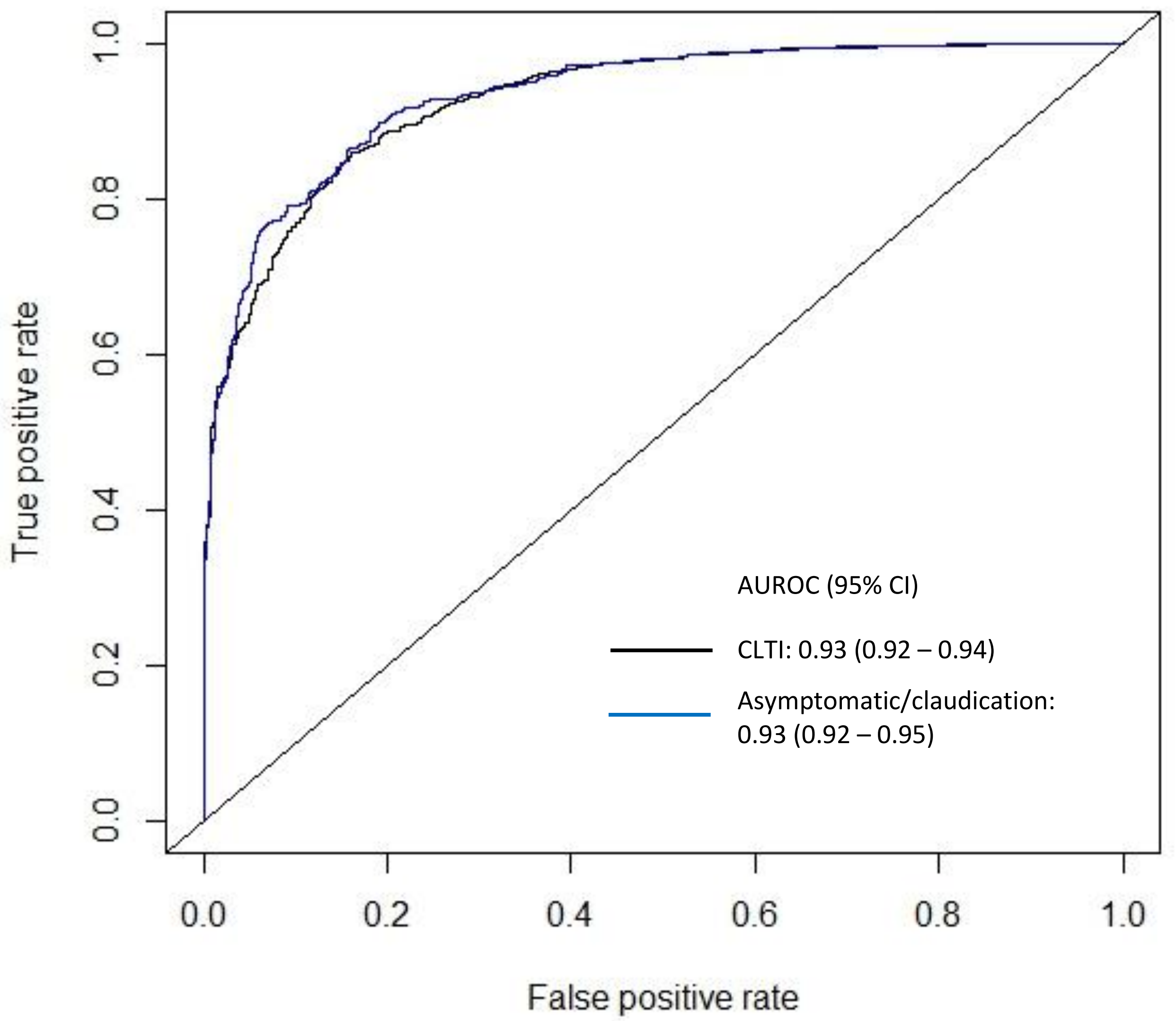

Supplementary Figure 6. Receiver operating characteristic curve for Extreme Gradient Boosting (XGBoost) model in predicting 30-day major adverse limb event or death following endovascular aortoiliac revascularization with subgroup analysis based on symptom status. AUROC (area under the receiver operating characteristic curve), CI (confidence interval).

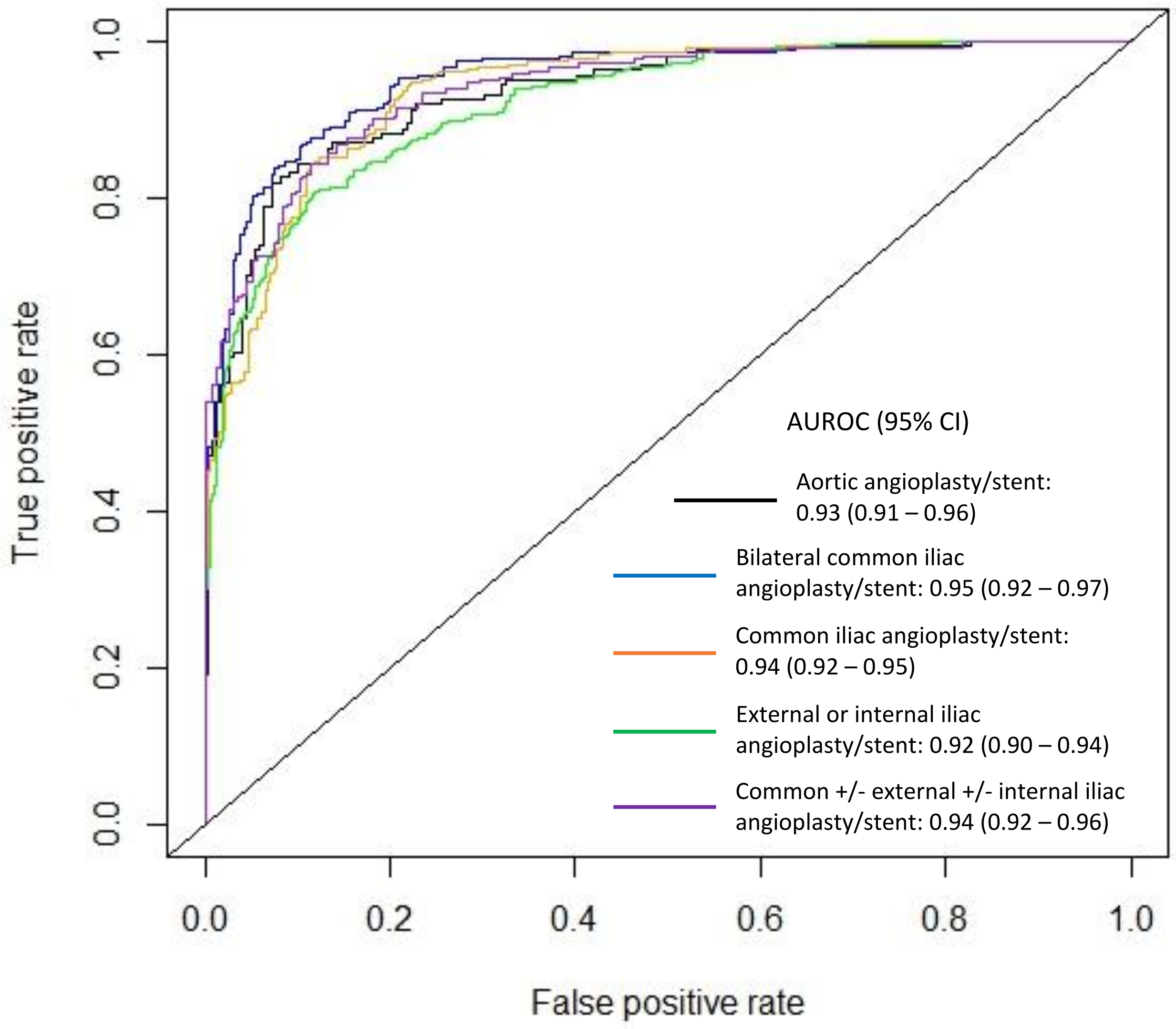

Supplementary Figure 7. Receiver operating characteristic curve for Extreme Gradient Boosting (XGBoost) model in predicting 30-day major adverse limb event or death following endovascular aortoiliac revascularization with subgroup analysis based on procedure type. AUROC (area under the receiver operating characteristic curve), CI (confidence interval).

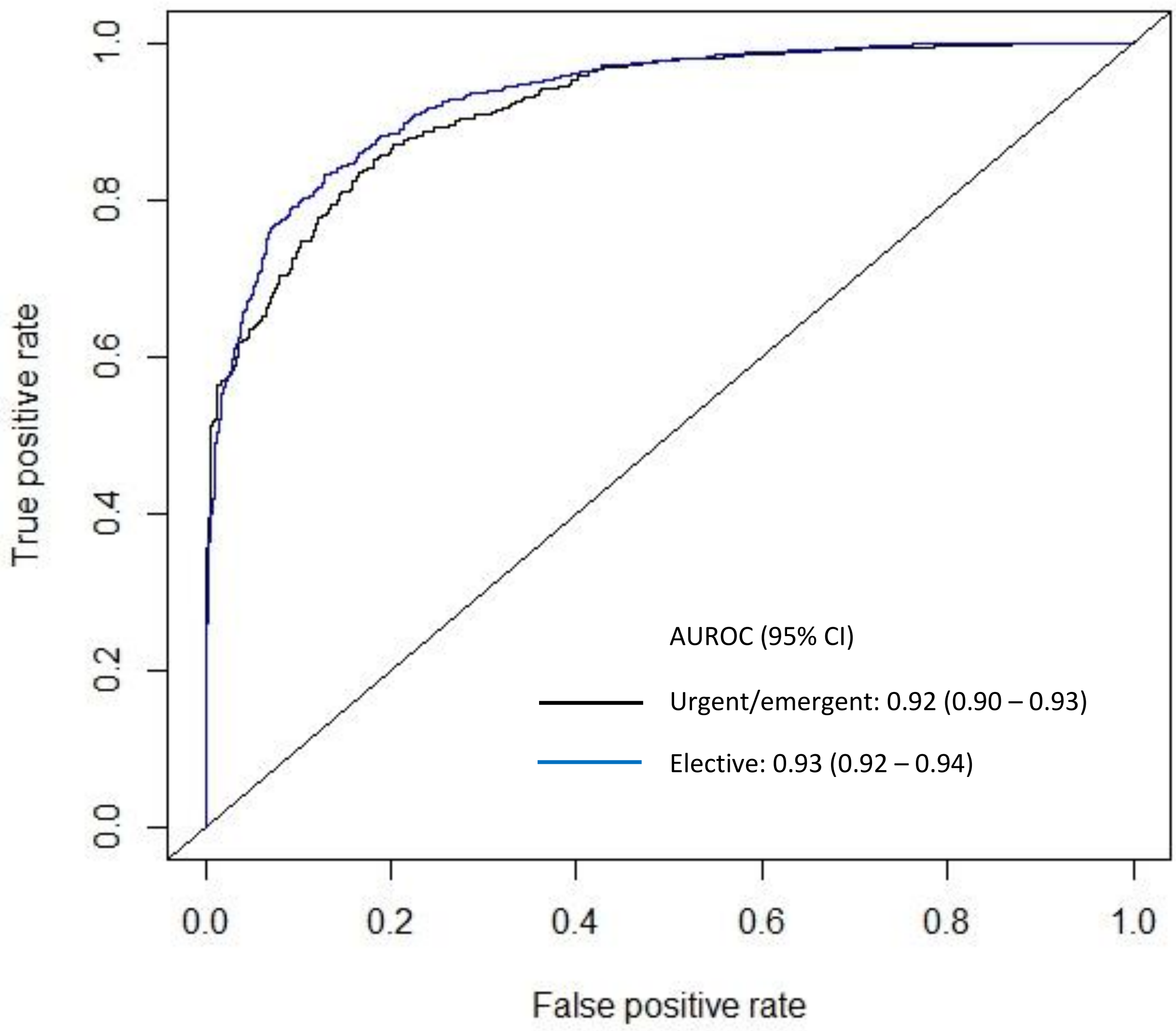

Supplementary Figure 8. Receiver operating characteristic curve for Extreme Gradient Boosting (XGBoost) model in predicting 30-day major adverse limb event or death following endovascular aortoiliac revascularization with subgroup analysis based on urgency. AUROC (area under the receiver operating characteristic curve), CI (confidence interval).

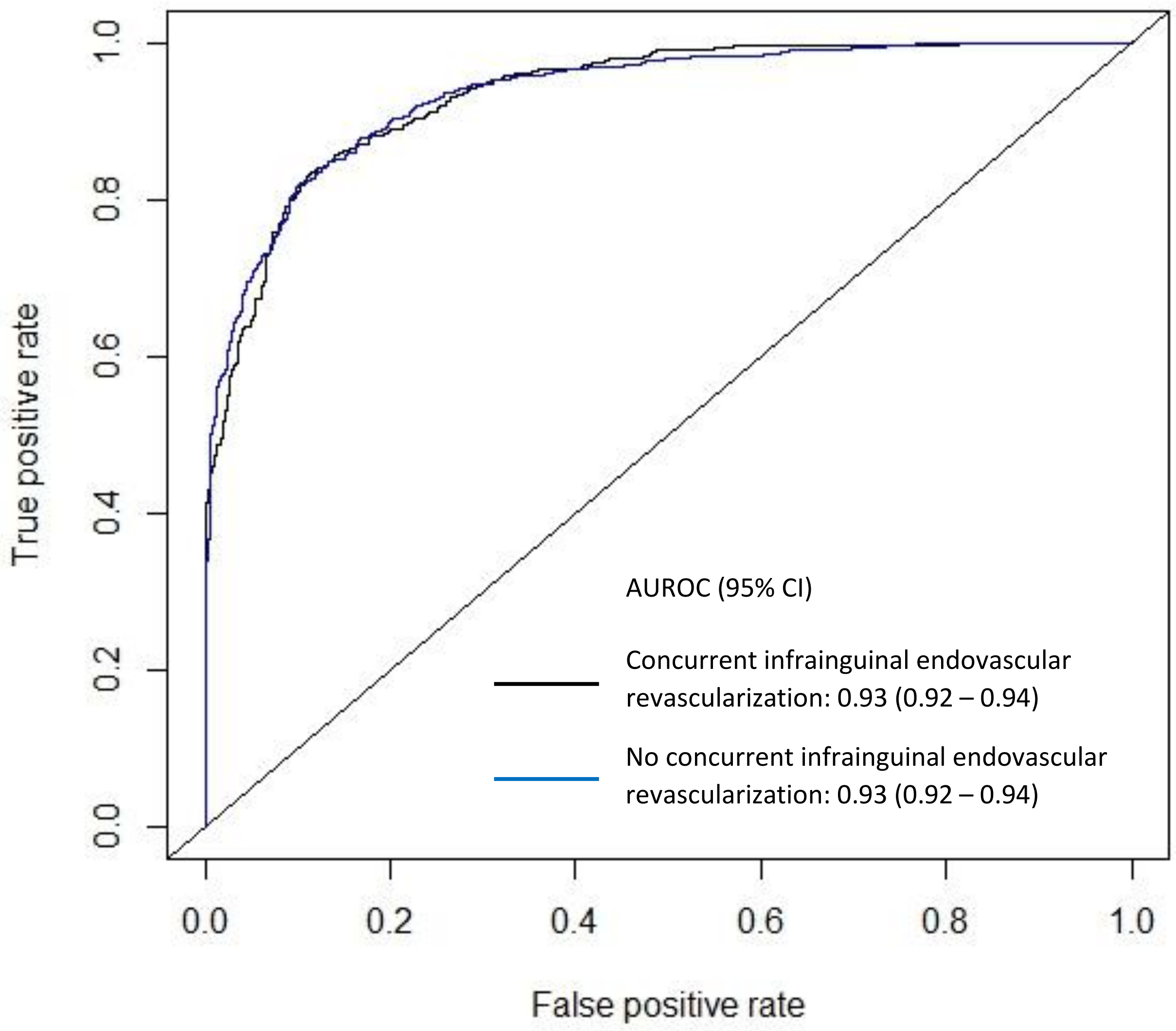

Supplementary Figure 9. Receiver operating characteristic curve for Extreme Gradient Boosting (XGBoost) model in predicting 30-day major adverse limb event or death following endovascular aortoiliac revascularization with subgroup analysis based on concurrent infrainguinal endovascular revascularization. AUROC (area under the receiver operating characteristic curve), CI (confidence interval).
